# A prognostic model based on prognosis-related ferroptosis genes for patients with acute myeloid leukemia

**DOI:** 10.3389/fmolb.2023.1281141

**Published:** 2023-12-11

**Authors:** Feima Wu, Guosheng Xu, Guangchao Li, Zhao Yin, Huijuan Shen, Kaiheng Ye, Yangmin Zhu, Qing Zhang, Ruiming Ou, Shuang Liu

**Affiliations:** ^1^ Department of Hematology, Guangdong Second Provincial General Hospital, Guangzhou, Guangdong, China; ^2^ Department of Blood Transfusion, Guangdong Second Provincial General Hospital, Guangzhou, Guangdong, China

**Keywords:** AML, prognosis-related ferroptosis genes, nomogram, prognosis, PARP inhibitor

## Abstract

**Background:** Acute myeloid leukemia (AML) is a heterogeneous disorder with an unpredictable prognosis. Ferroptosis, the iron-dependent cell death program, could serve as an alternative for overcoming drug resistance. However, its effect on AML remains largely unclear.

**Methods:** We collected RNA sequencing data and relevant clinical information of AML patients from The Cancer Genome Atlas to construct a prognosis prediction model. Risk score was calculated with eight prognosis-related ferroptosis genes (PRFGs) discovered through univariate analysis and Least Absolute Shrinkage and Selection Operator (LASSO) Cox regression. A nomogram was constructed by incorporating LASSO risk score, age, and cytogenetic risk based on univariate/multivariate Cox regression.

**Results:** Of the 33 AML PRFGs identified from the TCGA-derived dataset, 8 genes were used to construct a gene signature to predict AML prognosis. Principal component analysis and heatmap showed significant differences between the low and high risk score groups. Next, LASSO risk score, age, and cytogenetic risk were incorporated into the nomogram to predict the overall survival (OS) of AML patients. According to survival analysis, patients with a low risk score had markedly increased OS as compared to those with a high risk score. Based on the results of Gene Ontology and Kyoto Encyclopedia of Genes and Genomes, the differences between the two risk groups showed a close relationship with immune-related pathways and membrane transportation. The analysis of tumor-infiltrating immune cells and immune checkpoints revealed that the immunosuppressive tumor microenvironment possibly facilitated different prognostic outcomes between the two groups. Gene expression analyses showed that the mRNA expression levels of PARP1 and PARP3 (PARPs) were closely related to the different clinical subgroups and the analyzed OS in AML patients. Finally, the PARP inhibitor talazoparib and the ferroptosis inducer erastin exerted a synergistic anti-proliferative effect on AML cells.

**Conclusion:** We constructed a nomogram by incorporating PRFGs, and the constructed nomogram showed a good performance in AML patient stratification and prognosis prediction. The combination of PARP inhibitors with ferroptosis inducers could be a novel treatment strategy for treating AML patients.

## Introduction

Acute myeloid leukemia (AML) is a heterogeneous hematologic tumor with the features of abnormal growth of myeloblasts or pro-granulocytes without physical differentiation. Over the past 10 years, tremendous progress has been made in the development of approaches for treating AML, such as hematopoietic stem cell transplantation; inhibitors targeting MCL-1, IDH2, and NPM1/FLT3-ITD mutations, epigenetic agents, antibody-based treatments, and cellular therapies. Consequently, the survival of patients with AML has significantly improved (Newell and Cook, 2021). Between 2017 and 2019, nine new drugs were approved by the FDA for AML patients. Despite these advances, older patients with AML show poor prognostic outcomes, with a long-term survival rate of <15% (Short et al., 2018). Because AML is highly heterogeneous, the treatment of this disease is challenging due to varying clinical features, including abnormal genetic and cytogenetic characteristics, and isolated factors such as coexisting diseases and physical conditions (Dohner et al., 2017). Hence, a prognosis prediction model that incorporates these well-recognized factors is important to precisely stratify the pretreatment risk and to implement clinical treatment decision-making.

Ferroptosis is a specific cell death program triggered by iron-dependent phospholipid peroxidation, and it is regulated by different cellular metabolic pathways such as iron metabolism, redox homeostasis, amino acid/lipid/sugar metabolism, and mitochondrial activity (Stockwell et al., 2017; Jiang et al., 2021). Recently, ferroptosis has received increasing attention, and considerable progress has been achieved in developing drugs targeting the regulatory mechanisms of ferroptosis in cancer cells (Lei et al., 2022). Several studies have investigated the involvement of ferroptosis in AML. Yu et al. (2015) showed that the ferroptosis inducer erastin not only inhibits AML cell growth but also enhances their sensitivity to chemotherapeutic drugs. Du et al. (2019) revealed that dihydroartemisinin specifically induced AML cells ferroptosis by modulating the activation of the AMPK/mTOR/p70S6k autophagy pathway activation. Yusuf et al. (2020) found that leukemic cells, rather than healthy myeloid cells, were dependent on the aldehyde dehydrogenase 3a2 enzyme for oxidizing long-chain aliphatic aldehydes to prevent cellular oxidative injury and synthetic lethality of ferroptosis inducers. Moreover, glutathione peroxidase-4 (GPX4) (Liu et al., 2023), TP53 (Cui et al., 2022), reactive oxygen species (ROS) metabolism (Du et al., 2020), and glutathione (GSH) metabolism (Wei et al., 2020) were found to be closely associated with the ferroptosis process in AML cells and with prognostic outcomes in AML patients. Additionally, numerous ferroptosis-related genes (FRGs) have been discovered, but these genes have shown inconsistent functions. Recently, the association of FRGs with the prognostic outcome of AML patients has been investigated. However, a prognosis prediction model incorporating the prognosis-related FRGs together with the clinical characteristics of AML patients is yet to be established. In the present study, machine learning was used to assess data from AML patients collected from public databases. We then established a prognostic signature by incorporating prognosis-related FRGs and used this signature to generate a prognosis prediction model for AML patients.

## Materials and methods

### Cell lines

AML cell lines MOLM-13, U937 and KG-1a were acquired from American Type Culture Collection (ATCC). Cells were cultured at 37°C and 5% CO_2_ in humidified incubator. Culture medium for MOLM-13 and U937 consisted of RPMI-1640 with 10% v/v fetal bovine serum and 1% v/v penicillin/streptomycin, while medium for KG-1a was IMDM with 20% v/v fetal bovine serum and 1% v/v penicillin/streptomycin.

### Data collection

RNA sequencing data and the related clinical information of AML patients were obtained from The Cancer Genome Atlas (TCGA) database (https://portal.gdc.cancer.gov/repository/). Expression profiles and clinical information of AML patients from two datasets (GSE71014 and GSE37642) were obtained from the Gene Expression Omnibus (GEO) database (https://www.genecards.org/).

### PRFG screening

Prognosis-related genes (PRGs) of AML patients were analyzed with “survival” package in R software (version 4.2.1). PRGs were identified based on the following criteria: hazard ratio (95% CI) > 1.0 and *p*-value ≤ 0.05. The “limma” package was used to analyze differentially expressed genes (DEGs). The threshold was set as log2 fold change > 1 and false discovery rate < 0.05. FRGs were collected based on FerrDB (http://www.zhounan.org/ferrdb/current/). The intersection genes of PRGs, DEGs, and FRGs were defined as PRFGs.

### Protein-protein interaction (PPI) network construction

PPI network of PRFGs was constructed with STRING web tool (https://string-db.org/) and visualized with Cytoscape (version 3.9.1). The parameters were set as follows: network type: full STRING network, meaning of network edges: confidence, active interaction sources: experiments, text mining, databases, co-expression, neighborhood, gene fusion, co-occurrence, minimum required interaction score = 0.4, max number of interactors to show: Query proteins only. The node scores of the PPI network were calculated with cytoHubba module of Cytoscape. The top 10 nodes rank by node score were defined as hub genes.

### Establishment and verification of the PFRG-based prediction model for AML patients

The “glmnet” package in R software was used to establish a PRFG signature by least absolute shrinkage and selection operator (LASSO)-penalized Cox regression (Liu et al., 2018). The lowest partial likelihood of deviance was used to determine the model penalty parameter (λ). The regression coefficient (β) of the LASSO model was linearly combined with gene expression to determine the prognostic risk score. AML patients were assigned to the high- and low-risk score groups according to the threshold. Principal component analysis (PCA) was performed using the “prcomp” function in R software according to the risk scores of the identified genes. The effect of the prognostic PRFG-based signature on prediction was analyzed through Kaplan–Meier survival analysis and time-dependent receiver operating characteristic (ROC) curves.

### Construction of the prognostic nomogram for AML patients

Based on the univariate/multivariate Cox regression analysis of the clinical features of AML patients, a nomogram was constructed using the R packages “survival” and “rms.” Based on the median nomogram risk scores, AML patients were assigned to the high or low-risk score groups. The accuracy of the nomogram was evaluated based on ROC curves and the concordance index (C-index).

### Gene Ontology and Kyoto Encyclopedia of Genes and Genomes analysis

Kyoto Encyclopedia of Genes and Genomes (KEGG) and Gene Ontology (GO) analyses of DEGs between the low- and high-risk score groups were conducted with “clusterProfiler” and “ggplot2” packages in R software (Subramanian et al., 2005; Yu et al., 2012). *p* < 0.05 was considered the significance level for the enriched pathways.

### Analysis of immune profiles

To analyze the immune status of each sample, we used Cell-type Identification By Estimating Relative Subsets Of RNA Transcripts (CIBERSORT) to calculate 22 tumor-infiltrating immune cell (TIIC) proportions in AML patients (Newman et al., 2015; Becht et al., 2016). We also used CIBERSORT to convert mRNA data to tumor-infiltrating non-cancer cell proportions in the tumor microenvironment with standard annotation files for organizing gene expression profiles. The list of immune checkpoint molecules was derived from (Fang et al., 2022). By querying the PubMed, thirty-two molecules out of the forty-seven immune checkpoint list that related to AML were further analyzed.

### CCK-8 assay and treatment combination analysis

Cell viability was determined by cell counting kit-8 (CCK-8) assay (Beyotime Technology, China). After the cells culturing for 48 h, 20 µL CCK-8 solution was added to each well, and the absorbance (OD) value was measured at 450 nm. The concentrations of erastin used in this study were set as around 40% inhibitory activity of AML cell lines. For synergize assay, the concentrations of talazoparib were set as serial concentration less than EC50 of AML cell lines. The combination effect of talazoparib and erastin at indicated concentration, combination index (CI), fraction affected (FA) levels were calculated by CompuSyn software using Chou-Talalay method with constant-ratio combinations. CI values less than 1, equal to 1, greater than 1 indicate synergistic, additive, or antagonistic effects, respectively.

### Cell migration assay

A total of 1 × 10^5^ cells were resuspended with 200 μL serum-free medium and seeded into the Transwell chamber (8 μm in diameter, Corning, United States), then the chambers were insert into a well with 500 μL culture medium containing corresponding concentrations of drugs. The plate was placed for 72 h incubation at 37°C. The migrated cells from the chambers were imaged with a microscope and the number was calculated.

### Statistical analysis

Continuous variables that exhibited a normal distribution were presented as the mean ± standard deviation, and comparisons between groups were examined using Student’s *t*-test. Kaplan–Meier survival analysis was used to estimate overall survival and Cox regression was used to compare survival differences between patient groups. Survival analysis was carried out with “survminer” and “survival” R packages. R software (Version 4.2.1) and GraphPad Prism 9 were adopted for data analysis. *p*-value < 0.05 stood for statistically significant.

## Results

### Discovery of PRFGs

**FIGURE 1:**
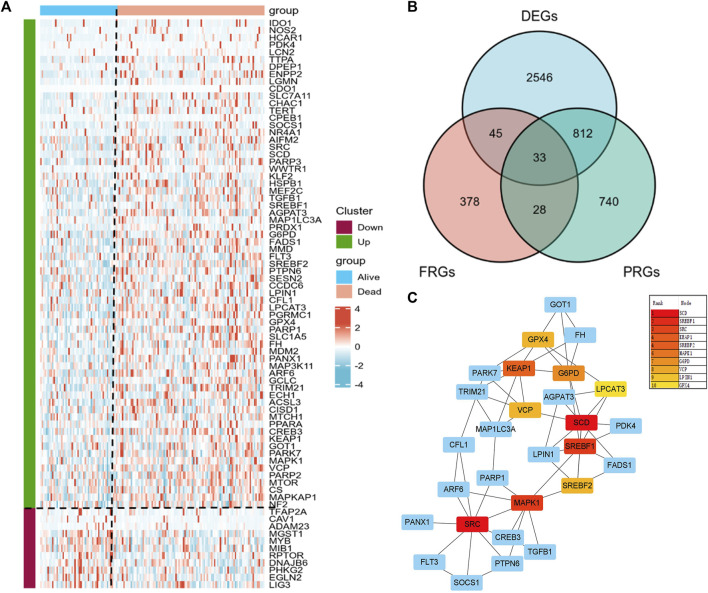
Prognosis-related gene (PRG) analysis in AML patients. **(A)** Heatmap of the differential expressed PRGs in AML patients. **(B)** Venn plots showing prognosis-related ferroptosis genes (PRFGs). **(C)** PPI network diagram of PRFGs.

We obtained 3436 DEGs and 1613 PRGs by comparing dead and alive AML patients derived from the TCGA database. Heatmap showed a total of 78 significant differential expressed FRGs in alive and dead patients and they were further divided into up- and downregulated groups according to their log2 fold-change (Figures 1A, B). By intersecting DEGs, PRGs, and FRGs, we obtained 33 PRFGs (Figure 1B). We then constructed protein–protein interaction (PPI) networks based on the STRING database to analyze and predict protein interactions and protein functional connectivity. Rank by node score of the PPI network, as shown in Figure 1C, SCD, SREBF1, SRC, SREBF2, KEAP1, etc. were identified as the hub genes.

### Establishment and verification of the prognostic gene signature

**FIGURE 2:**
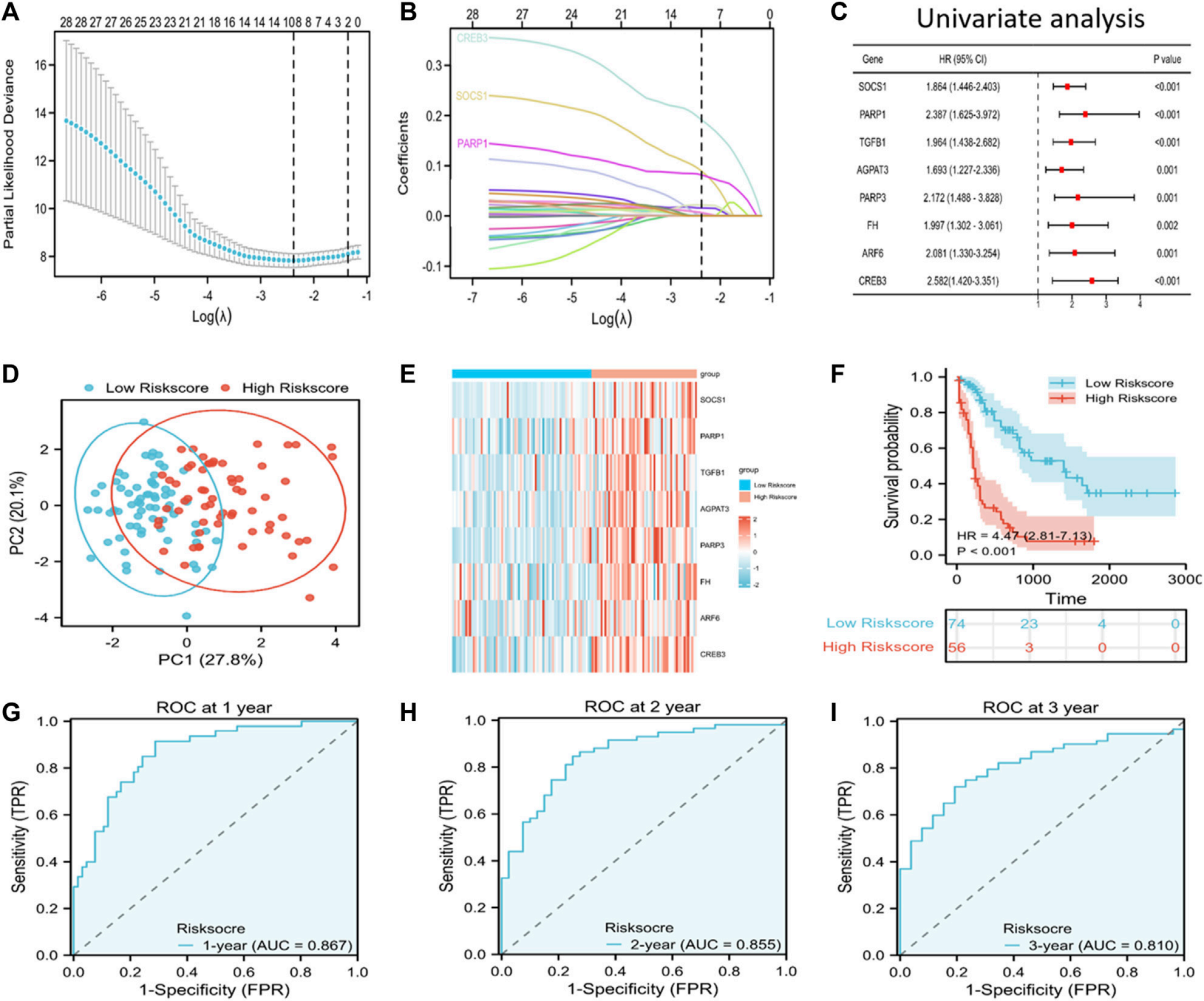
Discovery of prognosis-related ferroptosis genes (PRFGs) for establishing the prognosis prediction signature for AML patients derived from the TCGA database. **(A)** LASSO Cox regression of the PRFGs. **(B)** LASSO coefficients of the PRFGs. **(C)** Univariate Cox regression confirmed the relationship between PRFGs and the prognostic outcome for AML patients. **(D)** PCA plot showing AML cases based on the expression levels of the signature genes in both risk score groups. **(E)** Heatmap showing the mRNA levels of the eight chosen PRFGs in the low-risk score and high-risk score groups. **(F)** Kaplan–Meier survival curves suggest increased OS in the low-risk score group as compared to that in the high-risk score group. **(G–I)** ROC curves of the LASSO model to predict 1-, 2- and 3-year OS of AML patients.

We incorporated the 33 PRFGs in LASSO Cox regression (Figure 2A) and constructed the 8-gene signature according to the optimum λ value (Figure 2B). These 8 genes included *SOCS1*, *PARP1*, *TGFB1*, *AGAPT3*, *PARP3*, *FH*, *ARF6*, and *CREB3*. To clarify the association of the selected genes with patient survival, univariate Cox regression was performed (Figure 2C). According to the β-value of every gene discovered based on LASSO Cox regression, the prognostic risk score was calculated as follows: (0.0893* SOCS1 expression) + (0.0815 * PARP1 expression) + (0.0014 * TGFB1 expression) + (0.00159 * AGPAT3 expression) + (0.0065 * PARP3 expression) + (0.0217 * FH expression) + (0.0067 * ARF6 expression) + (0.1914 * CREB3 expression). Based on the median risk score, we assigned the patients to the high-risk score group (*n* = 74) or the low-risk score group (*n* = 56). Furthermore, based on the PCA results, patients in both subgroups showed a discrete distribution (Figure 2D). The expression levels of the selected genes also showed a significant difference between both groups (Figure 2E; Supplementary Figure S3). Moreover, Kaplan–Meier survival analysis indicated a significantly increased OS in the low-risk score group as compared to that in the high-risk score group (Figure 2F). Furthermore, the area under the ROC curve (AUC) values for 1-, 2-, and 3-year OS were 0.867, 0.855, and 0.810, respectively, which indicated good predictive performances of LASSO analysis (Figures 2G–I).

Next, GEO-derived data were analyzed for model validation. The AML patients from the GEO cohort were classified into the high or low-risk score group according to the risk score. A significant difference was noted between both subgroups, with a markedly increased survival rate in the low-risk score group compared to that in the high-risk score group (*p* < 0.001) (Supplementary Figures S1A–F, S2A–F).

### Risk score independently predicts the prognosis of AML

**FIGURE 3:**
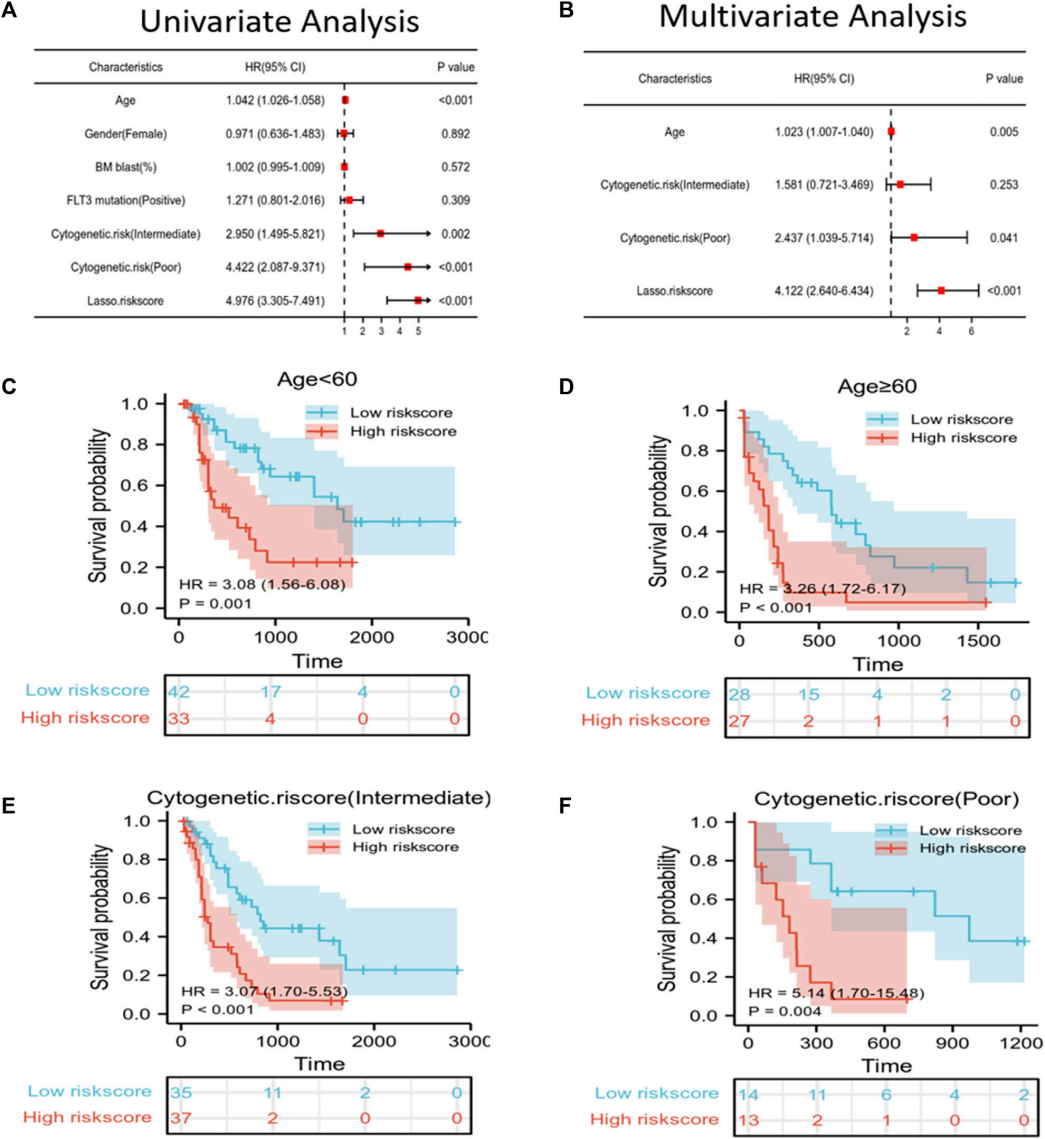
Independent prognostic factors of risk scores and clinical features. **(A,B)** Univariate/multivariate Cox regression confirmed that age, cytogenetic risk (poor), and LASSO risk score could independently predict prognosis. **(C–F)** Kaplan–Meier survival curves suggested that OS was associated with age < 60 **(C)**, age ≥ 60 **(D)**, cytogenetic risk score (intermediate) **(E)**, and cytogenetic risk score (poor) **(F)**.

To predict the OS of AML patients, the risk score and clinical features, including age, gender, BM blast percentage, FLT3 mutation, and cytogenetic risk were incorporated into the univariate/multivariate Cox regression. The results revealed that the risk score independently predicted patient survival. Furthermore, age, cytogenetic risk (intermediate), and cytogenetic risk (poor) also independently predicted the prognosis of *p* ≤ 0.05 (Figures 3A, B). All patients were then classified according to age, cytogenetic risk (intermediate), and cytogenetic risk (poor). Based on the Kaplan–Meier survival curves, the low-risk score group showed increased survival as compared to the high-risk score group (Figures 3C–F).

### Establishment and verification of the prognosis prediction nomogram

**FIGURE 4:**
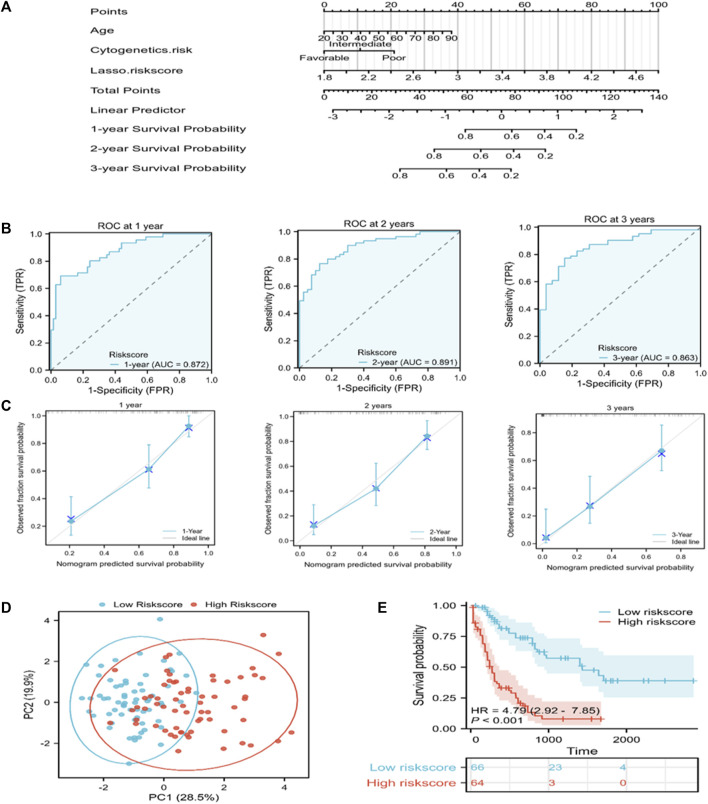
A nomogram for predicting the prognosis of TCGA-derived AML patients. **(A)** A nomogram was constructed to predict 1-, 2-, and 3-year OS in AML patients. **(B)** ROC curves for the nomogram for predicting 1-, 2-, and 3-year survival of AML patients. **(C)** Nomogram calibration curves showing survival probabilities at 1-, 2-, and 3-year. **(D)** PCA plot for AML cases according to the mRNA levels of the signature genes in both risk groups. **(E)** Kaplan-Meier survival curves suggest increased OS of patients in the low-risk score group as compared to that of patients in the high-risk score group.

A nomogram that can visually represent the prognosis prediction model was constructed by incorporating age, cytogenetic risk, and the risk score for illustrating patient survival (Figure 4A). The nomogram showed that the Lasso risk score most significantly affected 1-, 2-, and 3-year survival of AML patients, followed by cytogenetic risk and age. ROC curves and C-index were then used to evaluate the discrimination performance of the nomogram. The C-index value for predicting 1-, 2-, and 3-year patient OS was 0.785 (0.762–0.808). The area under the ROC curve (AUC) values for 1-, 2-, and 3-year OS were 0.872, 0.891, and 0.863, respectively, which were superior to those of the Lasso risk score model (Figure 4B). The model calibration performance was analyzed with a calibration curve. We found that our predicted results were consistent with the observed results (Figure 4C). The patients were assigned to high- or low-risk score groups in accordance with the median risk score value. Compared to the high-risk AML patients, low-risk AML patients showed markedly distinct dispersion direction with superior OS (*p* < 0.001) (Figures 4D, E).

### Functional annotation

**FIGURE 5:**
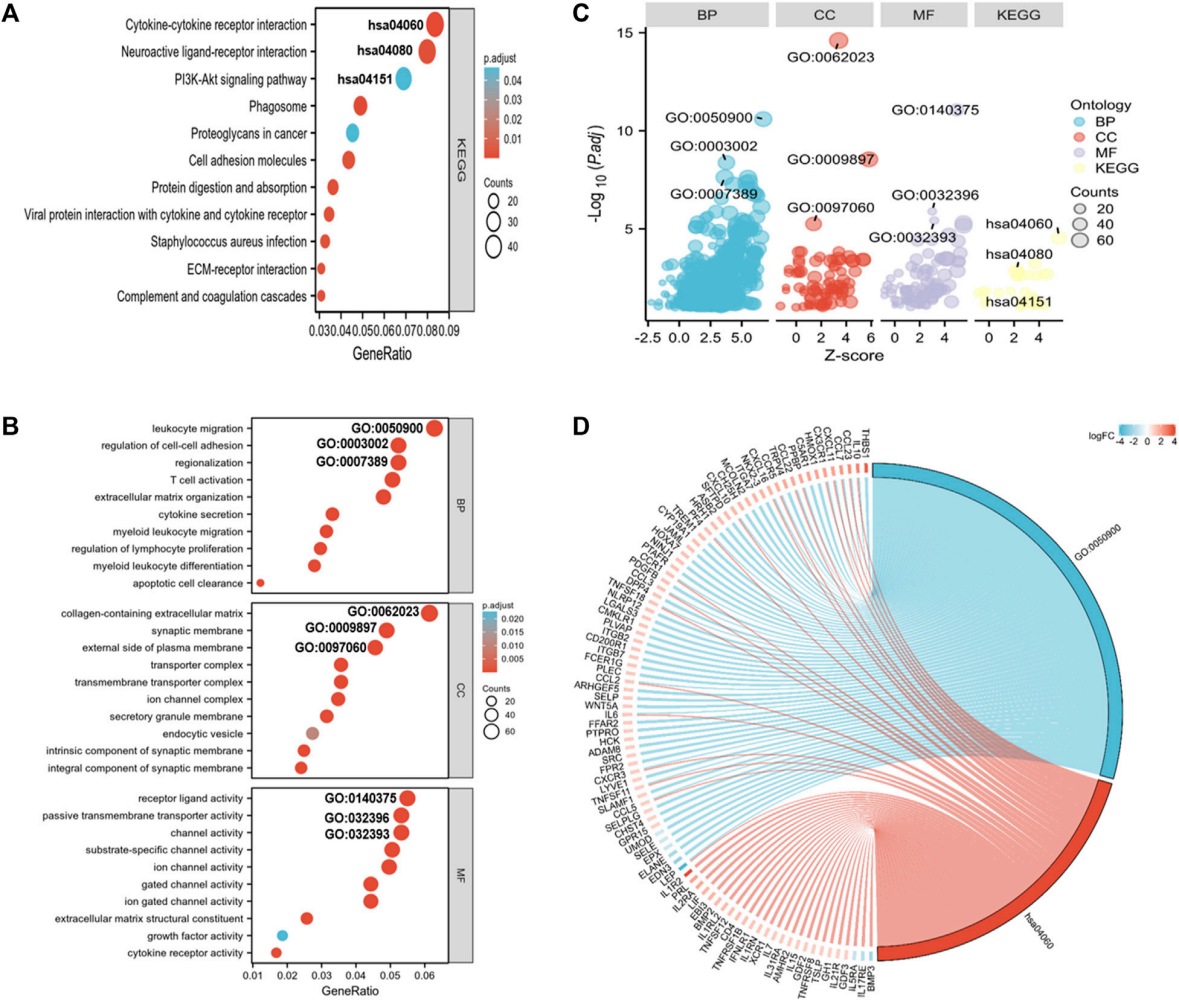
GO and KEGG analyses for DEGs in both risk score groups. **(A)** KEGG analysis of DEGs in both risk score groups. **(B)** GO annotation of DEGs in both risk score groups. **(C)** Bubble plots of top 3 GO and KEGG enrichment terms in both risk score groups. **(D)** Chord diagram showing the related genes of the GO term 0050900 (leukocyte migration) and the KEGG term hsa0460 (cytokine-receptor interaction).

To elucidate the pathways related to the nomogram risk score, KEGG pathway enrichment analysis was performed for DEGs in both risk score groups. We observed that DEGs were mostly associated with “neuroactive ligand-receptor interaction,” “cytokine-cytokine receptor interaction,” “PI3K-Akt pathway,” and “Phagosome” (Figure 5A). GO functional enrichment analyses showed that the biological process (BP) terms were mostly “leukocyte migration,” “regionalization,” and “regulation of cell-cell adhesion” (Figures 5B–D). The cellular component (CC) terms were mainly “collagen-containing extracellular matrix,” “synaptic membrane,” and “external side of plasma membrane” (Figures 5B–D). The molecular functions (MF) terms were “receptor ligand activity,” “passive transmembrane transporter,” and “channel activity” (Figures 5B–D).

### Immune status of AML patients based on the nomogram risk score groups

**FIGURE 6:**
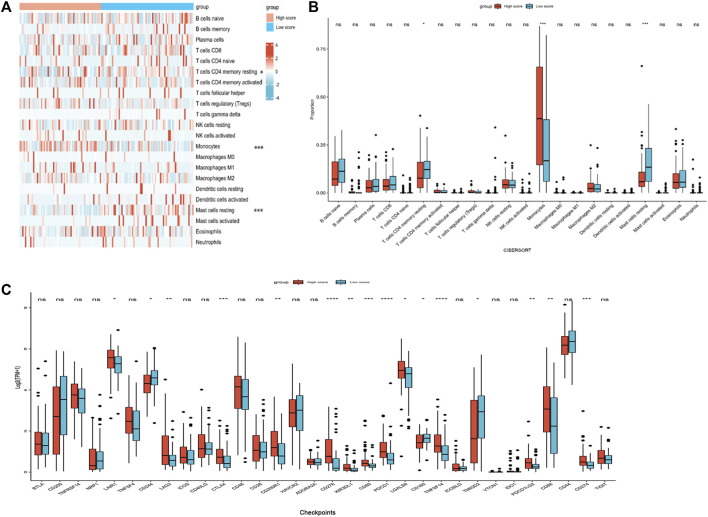
Immune profiles between the nomogram risk groups. **(A)** Heatmap showing immune cell expression between the risk score groups. **(B)** Comparison of diverse immune cell subtypes of both risk score groups. **(C)** Expression levels of immune checkpoint molecules in both risk score groups. *p < 0.05; **p < 0.01; ***p < 0.001; and ****p < 0.0001.

Because the enrichment analysis highlighted immune-related terms like “cytokine-cytokine receptor interaction” (hsa04060) and “leukocyte migration” (GO005090) (Figures 5A, B, D), we examined the correlation between the nomogram and TIICs. The differences of 22 types of TIICs in AML patients between the two risk score groups were assessed by the CIBERSORT algorithm. Figure 6A shows the similarities and differences in immune cell infiltration between the AML subgroups. “T cells CD4^+^ memory resting,” “Mast cell resting,” and “Monocyte” exhibited significant differences between both risk score groups (Figure 6B). Immune checkpoint molecules are the indicators for prognosis and serve as immunotherapeutic targets for AML patients. The expression levels of *LAIR1*, *LAG3*, *CTLA4*, *CD200R1*, *CD276*, *KIR3DL1*, *CD80*, *PDCD1*, *LGALS9*, *TNFSF14*, *PDCD1LG2, CD86*, *and CD274* in high-risk patients were markedly increased as compared to those in low-risk patients (Figure 6C). We further evaluated the association between immune checkpoint molecules and OS in AML patients. Higher expression levels of *LAIR1*, *CD276*, *LGALS9*, *PDCD1*, *PDCD1L2G*, and *TNFSF14* levels were correlated with poor OS (Supplementary Figure S4). Taken together, these data suggest that the poor prognostic outcome in the high-risk score group may be partly associated with the tumor immune microenvironment.

### Correlation between the expression levels of the risk-associated genes and clinicopathological subgroups of AML patients

**FIGURE 7:**
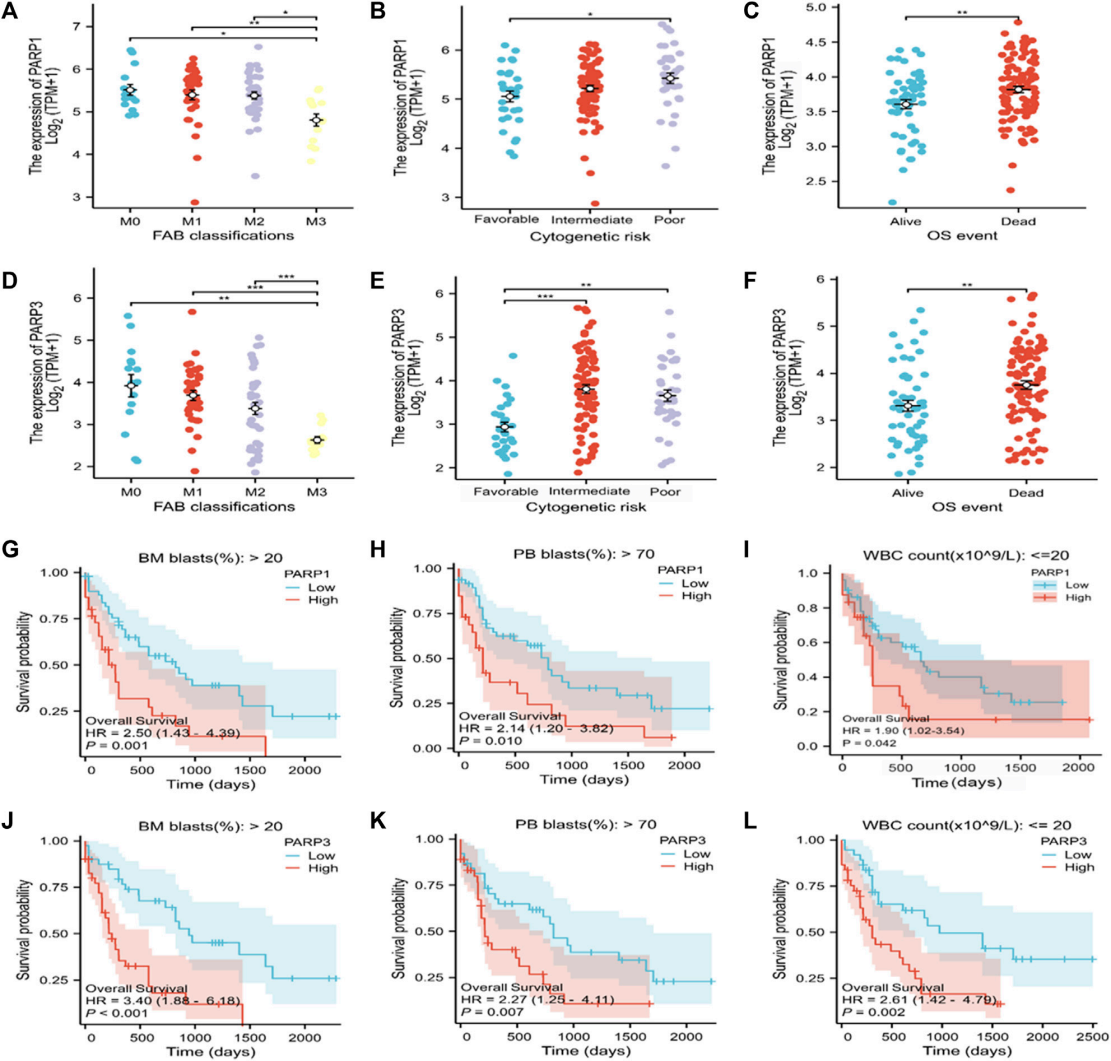
Association of the expression of PARPs with different clinical features of AML. **(A–C)** Correlation between PARP1 expression and FAB classification **(A)**, cytogenetic risk **(B)**, and OS event **(C)**. **(D–F)** Correlation between PARP3 expression and FAB classification **(D)**, cytogenetic risk **(E)**, and OS event **(F)**. **(G–I)** Correlation between PARP1 expression level and BM blasts (20%) > 20% clinical subgroup, PB blasts (20%) > 70% clinical subgroup, and WBC count (× 10^9^/L) ≤ 20. **(J–L)** Correlation between PARP3 expression level and BM blasts (20%) > 20% clinical subgroup, PB blast (20%) > 70% clinical subgroup, WBC count (× 10^9^/L): ≤20. *p < 0.05; **p < 0.01; and ***p < 0.001. OS, overall survival; BM, bone marrow; WBC, white blood cell; PB, peripheral blood.

PARPs are potential therapeutic targets for AML. However, the correlation between PARPs expression levels and AML clinicopathologic features, as well as the prognosis of patients with specific clinical variables remained to be explored. PARP1 and PARP3 were selected for further investigations as they showed significantly higher expression levels in the high Lasso risk score groups in all the analyzed datasets (Figure 2E; Supplementary Figures S1B, S2B, S3). According to the subgroup classification, the association between the clinicopathologic features of TCGA AML patients and the expression level of PARPs was analyzed. As shown in Figures 7A–F, the expression levels of PARP1 and PARP3 were significantly associated with the clinicopathologic features of FAB classification, cytogenetic risk, and OS event. Moreover, a higher expression level of PARP1 or PARP3 also predicted poor prognosis in the clinical subgroups of BM (bone marrow) blasts (%) > 20, PB (peripheral blood) blasts (%) > 70, and WBC (white blood cell) count (×10^9^/L) ≤ 20 (Figures 7G–L).

### The PARP1 inhibitor talazoparib shows a synergistic effect with the ferroptosis inducer erastin on AML cells

**FIGURE 8:**
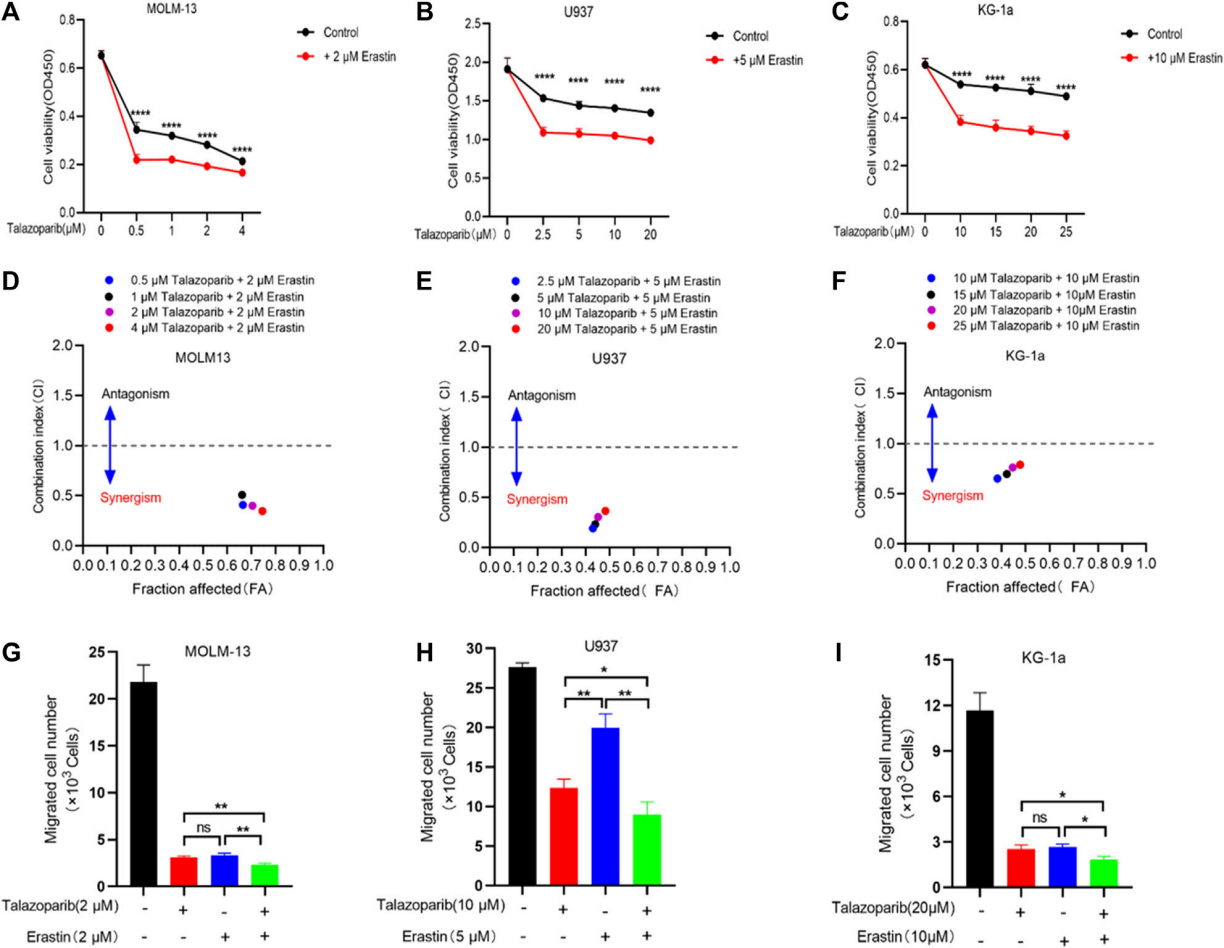
Erastin synergistically sensitizes AML cells to talazoparib. **(A–C)** Cell viability in MOLM-13 **(A)**, U937 **(B)**, and KG-1a **(C)** cells exposed to talazoparib and/or erastin treatment at specific doses. **(D–F)** Chou-Talalay plot showing the synergistic effects of specific treatments in MOLM-13 **(D)**, U937 **(E)**, and KG-1a **(F)** cells. Purple/black/red/blue dots in the plot represent talazoparib combined with erastin at specific doses. CI values of <, = , and >1 represent synergistic, additive, and antagonistic effects, respectively. **(G–I)** The synergistic effects of specific treatments in inhibiting MOLM-13 **(G)**, U937 **(H)**, and KG-1a **(I)** cells migration. *p < 0.05; **p < 0.01; ***p < 0.001; and ****p < 0.0001.

Antitumor drug combinations can effectively prevent resistance and provide novel treatments. Previous studies have shown that the PARP inhibitor shows a synergistic effect with ferroptosis inducers on BRCA-proficient ovarian cancer. We tested whether this drug synergy also affected the survival of AML cells. Consistent with the results of previous studies, talazoparib inhibited the growth of AML cells (Figures 8A–C). Furthermore, every combination index at specific talazoparib and erastin doses was <1 for MOLM-13, U937, and KG-1a cells (Figures 8D–F). Talazoparib also showed a synergistic effect with erastin to inhibit AML cell migration (Figures 8G–I; Supplementary Figure S5). These findings suggest that the PARP1 inhibitor shows a synergistic effect with the ferroptosis inducer erastin for inhibiting the growth and migration of AML cells.

## Discussion

In the present study, to predict the survival of AML patients, a prognosis prediction model was constructed based on the combination of 8 prognosis-related FRGs and clinical characteristics. The established model exhibited favorable calibration and discrimination performance for predicting patient survival. The association of the model with TIICs and immune checkpoint molecules was also partially investigated. The PARP inhibitor talazoparib showed a synergistic effect with the ferroptosis inducer erastin to enhance anti-proliferation efficacy for AML cells. AML has the highest occurrence frequency among acute leukemias during adulthood. Presently, cytogenetic markers play a critical role in stratifying the associated risk and treatment of AML patients. Although several studies have been conducted to find appropriate prognostic biomarkers, AML remains the disease with substantially different prognostic outcomes. The 5-year OS rate of AML is <50%, and the 2-year survival rate is only 20% among old people with AML (Gregory et al., 2009; Riva et al., 2012). Ferroptosis is a novel cell death program (Dixon et al., 2012), which is closely associated with AML (Yin et al., 2022). Although some ferroptosis-related prediction models for AML have been reported (Cui et al., 2022; Jinghua Wang et al., 2022; Kai Zhu et al., 2022; Yu et al., 2022), a model that incorporates well-recognized factors is highly important to precisely stratify AML patients.

By analyzing DEGs in AML patients, 33 prognosis-related ferroptosis DEGs were obtained based on 3436 DEGs in dead and alive patients. We chose eight genes following LASSO Cox regression and univariate analysis. Most of these genes were verified or predicted to be closely associated with cancers. The increased SOCS1 level in the bone marrow of AML patients was closely associated with advanced age, mutations in *FLT3-ITD*, *NPM1*, and *DNMT3A*, and SOCS1 overexpression in zebrafish mimic leukemia phenotype (Hou et al., 2017). PARPs, including PARP1 and PARP3, can mediate the early stage in DNA damage response. The inhibition of these proteins shows varying degrees of antitumor activity in AML, which mainly depend on the rearrangement of the genes (Padella et al., 2022). TGFB1 induces ALDH^+^ stem cell-like phenotype in AML cells and contributes to leukemogenesis and chemotherapy resistance (Yuan et al., 2020). ARF6 belongs to the small GTPase ADP-ribosylation factor (Arf) family, and the upregulation and activation of ARF6 are markedly associated with the migration and invasion of multiple cancers (Li et al., 2017). CREB3 has been identified as an HDAC3-interacting protein that enhances NF-κB activation and promotes the migration of breast cancer cells (Kim et al., 2010).

The clinical features of AML patients are closely associated with their prognostic outcomes. To optimize the model and improve its survival prediction performance, we selected age and cytogenetic risk based on univariate/multivariate Cox regression. Age and cytogenetic risk are significantly associated with AML performance status, multidrug resistance, and prognosis outcome (Appelbaum et al., 2006; Dohner et al., 2017); hence, we incorporated these two clinical features and a gene signature to construct the nomogram. Additionally, the ROC curve and C-index were used to evaluate the discrimination performance of the nomogram. The C-index for 1-year, 2-year, and 3-year OS of TCGA-derived AML patients was 0.785 (0.762–0.808), while the AUC values were 0.872, 0.891, and 0.863, respectively. These findings suggest the favorable discrimination performance of the model for survival prediction. Furthermore, according to the calibration curve, the constructed nomogram demonstrated good calibration. Based on the nomogram risk score, the patients were assigned to the high-risk score group or the low-risk score group. Functional annotation of the DEGs in both risk groups revealed that the enriched functional terms mainly involved leukocyte cell migration, T cell activation, and transmembrane transportation. We postulated that this finding might be correlated with the tumor-infiltrating cells in both subgroups. Hence, the proportions of immune cell in both groups were determined. CD4^+^ memory resting T cells and resting mast cells showed an evidently higher proportion in the low-risk group, while monocytes showed a higher abundance in the high-risk score group. These findings suggest the association of the poor prognostic outcome of AML patients with immune cell infiltration. FRGs also possibly affect cancer cells through immune cells. We compared the expression levels of the immune checkpoint molecules such as *LAIR1*, *LAG3*, *CTLA4*, *CD200R1*, *CD276*, *KIR3DL*, *CD80*, *PDCD1*, *LGALS9*, TNFSF14, *PDCD1L2G*, CD86, and *CD274* in high-risk and low-risk AML patients. AML patients showing a higher expression level of *LAIR1*, *CD276*, *LGALS9*, *PDCD1*, *PDCD1L2G*, and *TNFSF14* experienced worse prognosis; thus, indicating that targeting these immune checkpoints may be beneficial for treating high-risk AML patients.

PARP1 and PARP3 belong to the Ploy (ADP-ribose) polymerase superfamily and are associated with DNA damage response (DDR). Based on our computational results, PARP1 and PARP3 were found to be associate with certain clinicopathologic features and overall survival of clinical subgroups. These data may facilitate the risk grouping and treatment of AML patients. Moreover, inhibition of PARPs is suggested to enhance the antitumor effect by regulating ferroptosis. Hong et al. (2021) reported that PARP inhibitor olaparib synergizes with erastin via repressing SLC7A11 in BRCA-proficient ovarian cancer cells. The mechanism underlying this synergistic effect is that the repression of SLC7A11 by olaparib may potently enhance lipid peroxidation and ferroptosis. Another study conducted by Tang et al. (2022) showed that PARPs inhibitor olaparib enhances the arsenic trioxide induces ferroptosis by suppressing the expression levels of stearoyl-CoA desaturase1 (SCD1) in platinum-resistant ovary cancer cells. It is, therefore, rational to postulate that PARP1 inhibitors may show a synergistic effect with ferroptosis inducers. Our preliminary results show that the PARP1 inhibitor talazoparib exhibited a synergistic effect with the ferroptosis inducer erastin in suppressing the growth and migration of AML cells. To the best of our knowledge, the combination of talazoparib and erastin in AML has not been previously reported. Since PARPs inhibitors were reported to enhance the oxidative stress of cancer cells (Giovannini et al., 2019) and oxidative stress enhance the lipid peroxidative-dependent ferroptosis (Lei et al., 2022), the synergistic effect of talazoparib and erastin may be achieved by enhancing the ferroptotic cell death pathway.

In conclusion, we constructed a new prognosis prediction model involving 8 PFRGs and clinical characteristics. The model showed favorable calibration and discrimination performance. Our study also provided preliminary evidence that ferroptosis inducers sensitize AML cells to PARP inhibitors, which may benefit AML treatment.

## Data Availability

The original contributions presented in the study are included in the article/Supplementary Material, further inquiries can be directed to the corresponding authors.
