# Supplementary material for: A prognostic model based on prognosis-related ferroptosis genes for patients with acute myeloid leukemia
Source: Front Mol Biosci. 2023 Dec 11;10:1281141. doi: 10.3389/fmolb.2023.1281141 (PMC10754970; doi:10.3389/fmolb.2023.1281141)
Supplement: Supplementary file 1 [file Table1.DOCX]

Supplemental figures


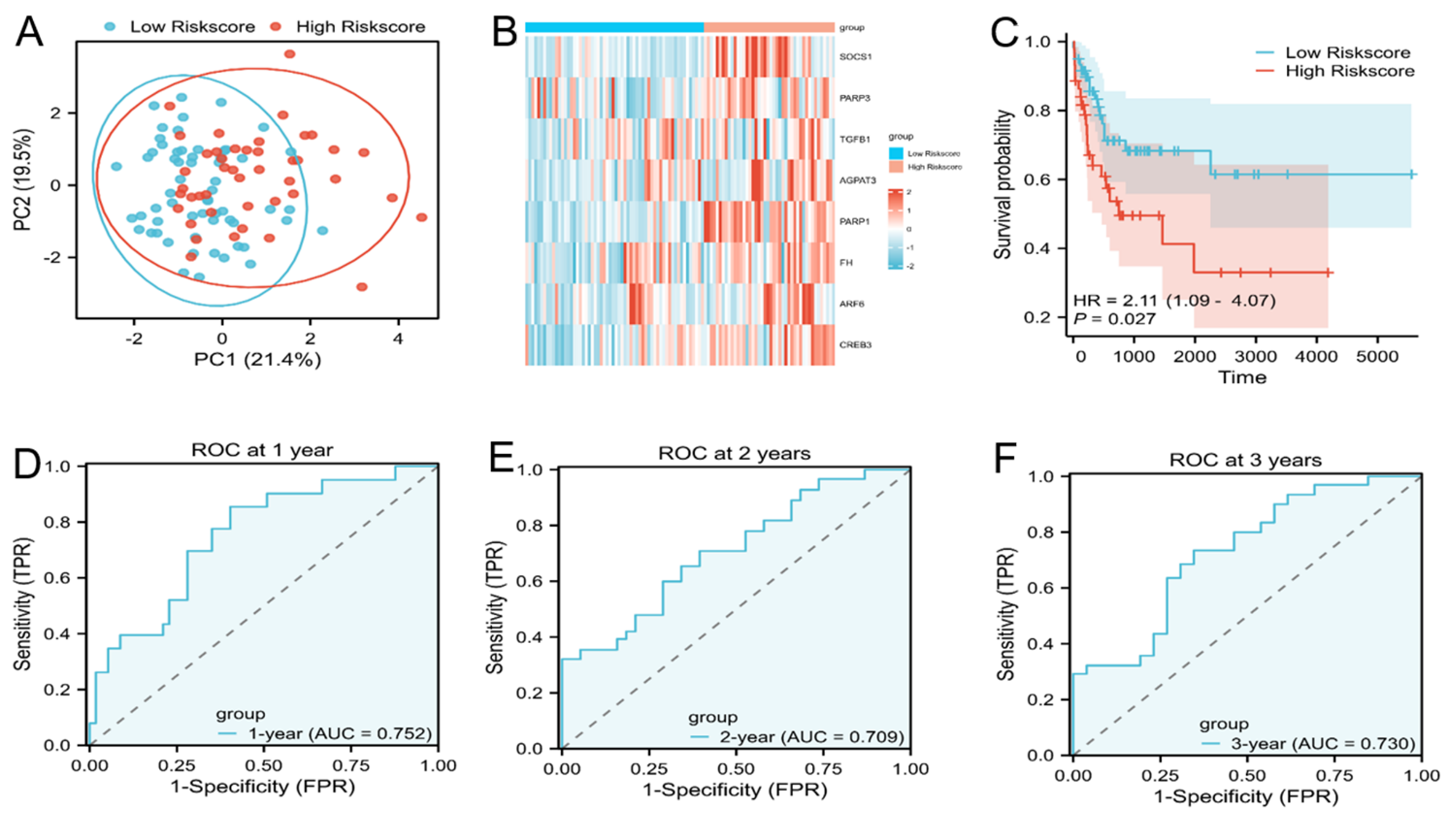


**Supplementary Figure 1**. Evaluation of the PRFG signature for the GSE71074 cohort. (A) PCA plot for AML patients according to the expression levels of the signature genes in both risk score groups. (B) Heatmap showing the mRNA levels of the signature genes for both risk score groups. (C) Kaplan–Meier survival curves suggest increased OS of the low-risk score group as compared to that of the high-risk score group. (D-E) Risk score-based ROC curves to predict 1-, 2-, and 3-year OS of AML patients.


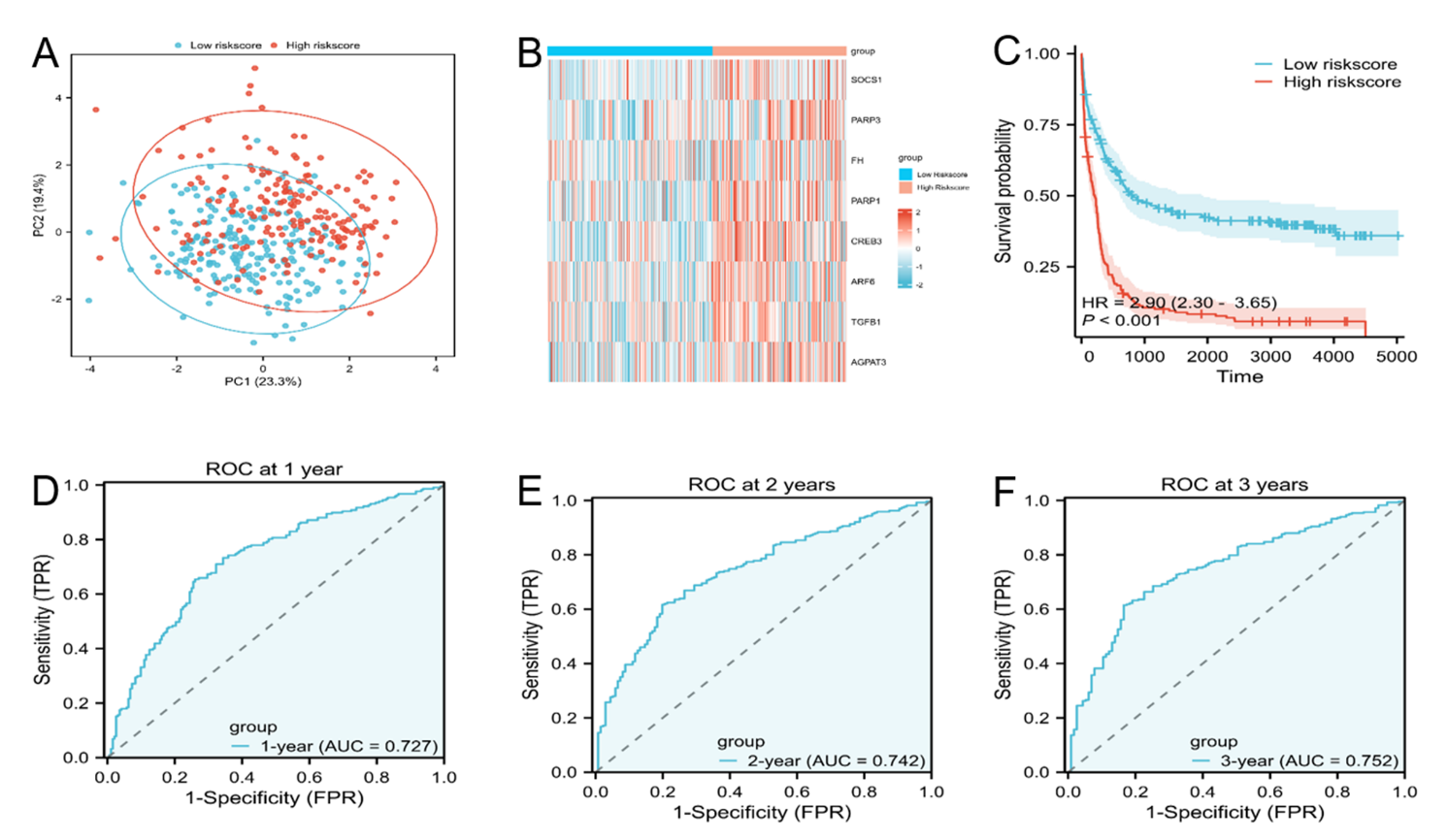


**Supplementary Figure 2.** Evaluation of the PRFGs signature for the GSE37642 cohort. (A) PCA plot for AML patients cases on the basis of the expression levels of the signature genes in both risk score groups. (B) Heatmap showing the mRNA levels of the signature genes in both risk score groups. (C) Kaplan–Meier survival curves suggest increased OS of the low-risk score group as compared to that of the high-risk score group. (D-E) Risk score-based ROC curves to predict 1-, 2-, and 3-year OS of AML patients.


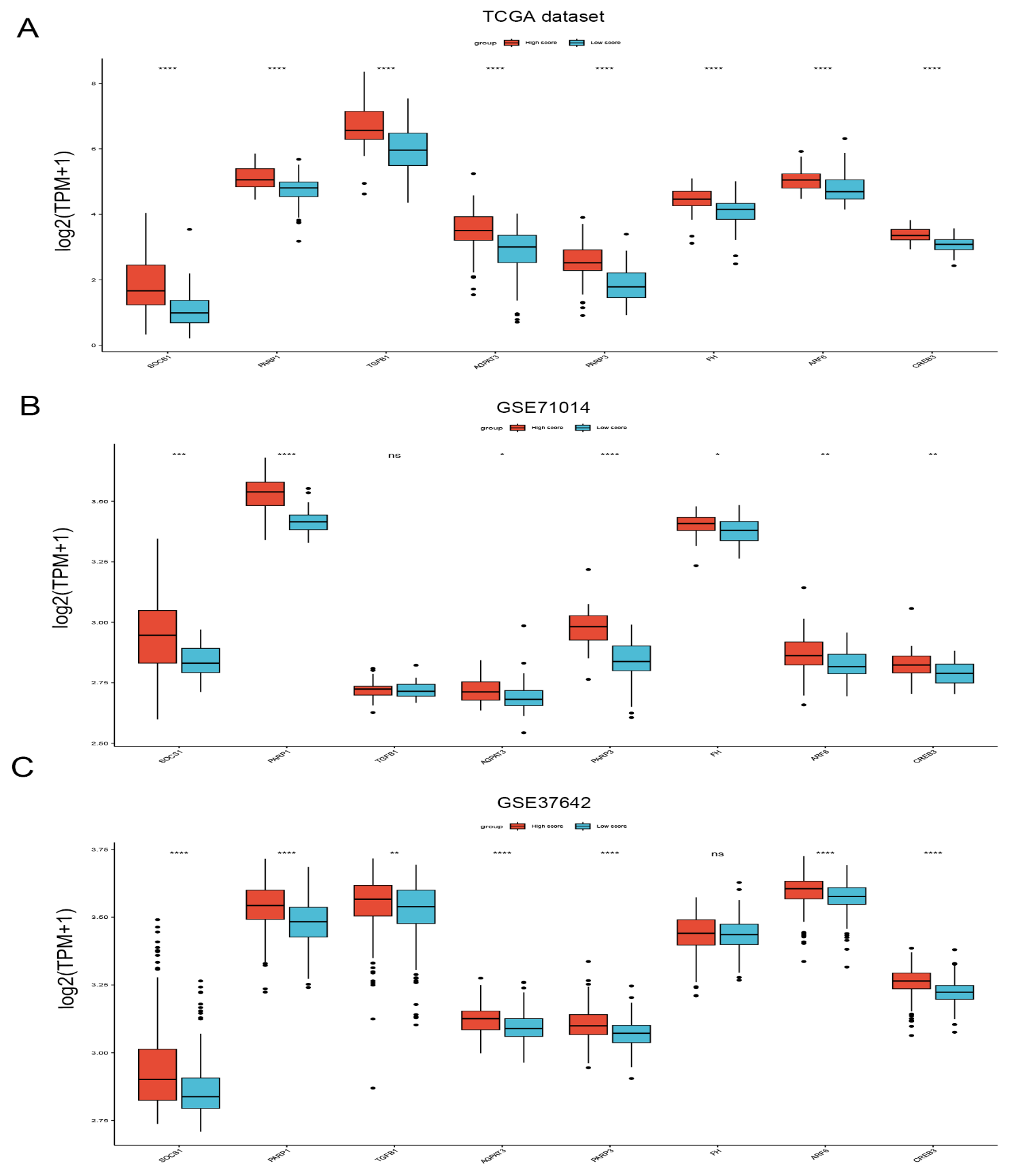


**Supplementary Figure 3.** Comparison of risk genes expression levels base on risk score grouping. (A-C) Box plot for comparison of risk gene expression levels of AML patients in TGCA (A), GSE71014 (B) and GSE 37642 (C) cohorts. ns, not significant;* p< 0.05, **p<0.01,***p<0.001,****p<0.0001.


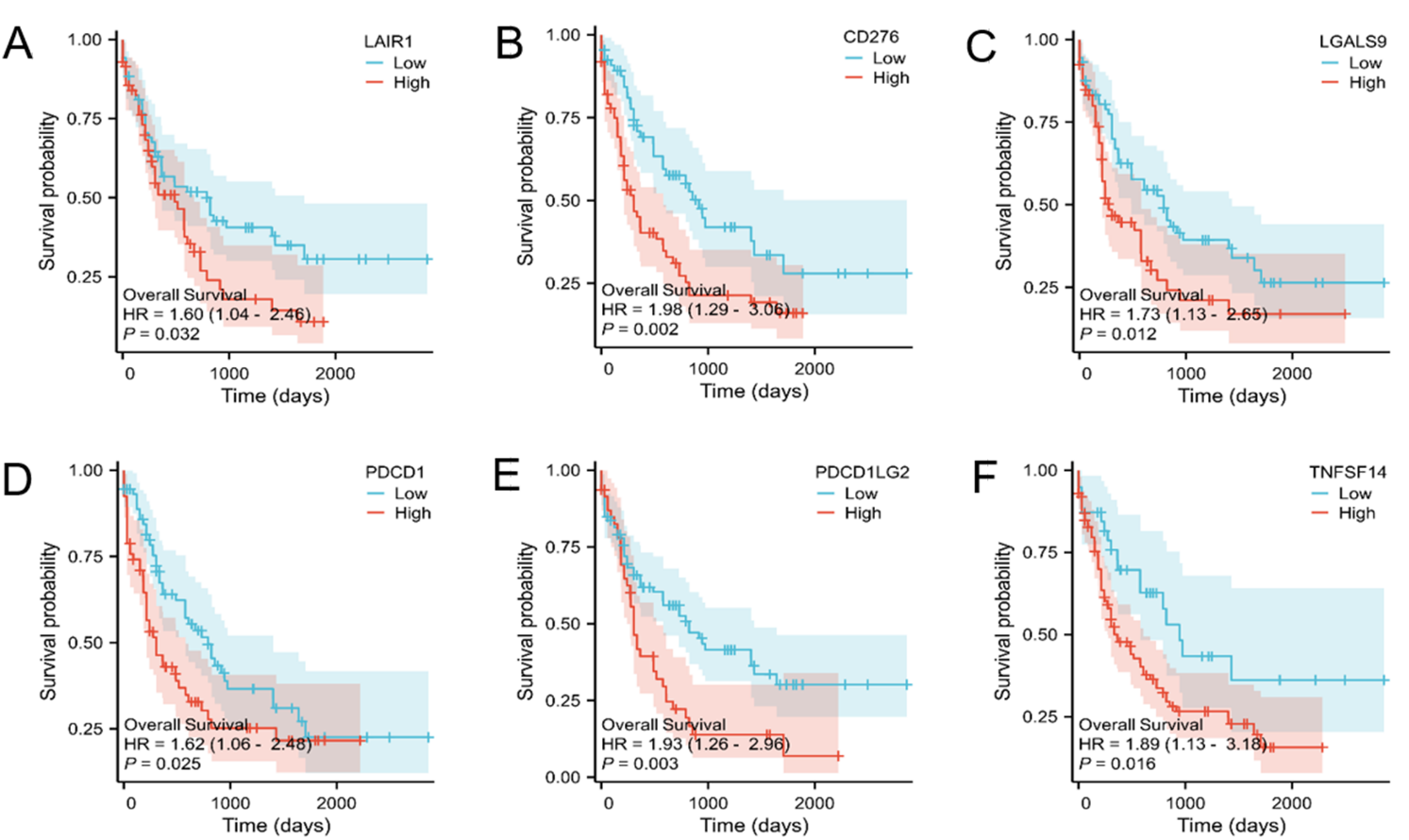


**Supplementary Figure 4.** Correlation between the expression levels of immune checkpoint molecules and overall survival. (A-F) Kaplan–Meier survival curves suggest increased OS of the low-risk score patients showing a low expression of *LAIR1* (A), *CD276* (B), *LGALS9* (C), *PDCD1* (D), *PDCD1LG2* (E), and *TNFSF14* (F) as compared to those in the high-risk score group.





**Supplementary Figure 5.** The migration assay of AML cell lines. (A-C)The bright filed images of migrated MOLM-13(A), U937 (B) and KG-1a (C) cells after indicated drugs treatments. Bar represent 400 μm.
